# 

**Published:** 1874-09

**Authors:** 


### Books and Pamphlets Received.

The Medical Register and Directory of the United States, systematically arranged by States. By S. W. Butler, M. D. Philadelphia: Office Medical and Surgical Reporter, 1874.

Erysipelas and Child-bed Fever. By Thomas C. Minor, M. D., Cincinnati. Robert Clarke & Co., 1874.

The Complete Handbook of Obstetric Surgery; Or Short Rules of Practice in every Emergency. By Charles Clay, M. D. Philadelphia: Lindsay & Blakiston, 1874.

Surgical Emergencies; together with Emergencies attendant on Parturition and the treatment of Poisoning. By Wm. Paul Swain, F.R.C.S. Philadelphia: Lindsay & Blakiston, 1874.

Transactions of the Medical Society of the State of Alabama, for 1874.

Transactions of the Michigan State Medical Society for 1874.

The Hypodemic use of Quinine: A Dangerous Experimental Medication and rarely justifiable. By Stephen Rogers, M. D., from New York Medical Journal, Sept., 1874.

THE BUFFALO

**Medical and Surgical Journal**

PUBLISHED MONTHLY,

Containing Original Articles, Reports of Medical Societies and Hospitals, Editorial, Reviews, Correspondence, Army News, etc., etc ; including the usual variety of Medical Periodical Publications. Specimen copies sent on application.

Address Buffalo Medical & Surgical Journal, Buffalo, N. Y.

TERMS OF SUBSCRIPTION, - - $3.00 PER YEAR, IN ADVANCE.

